# Supplementary material for: Multiomic QTL mapping reveals phenotypic complexity of GWAS loci and prioritizes putative causal variants
Source: Cell Genom. 2025 Feb 21;5(3):100775. doi: 10.1016/j.xgen.2025.100775 (PMC11960542; doi:10.1016/j.xgen.2025.100775)
Supplement: Document S1. Figures S1–S18 [file mmc1.pdf]

Cell Genomics, Volume *5*

Supplemental information

Multiomic QTL mapping reveals phenotypic complexity of GWAS loci and prioritizes putative causal variants

Timothy D. Arthur, Jennifer P. Nguyen, Benjamin A. Henson, Agnieszka D'Antonio-Chronowska, Jeffrey Jaureguy, Nayara Silva, iPSCORE Consortium, Athanasia D. Panopoulos, Juan Carlos Izpisua Belmonte, Matteo D'Antonio, Graham McVicker, and Kelly A. Frazer

SUPPLEMENTAL FIGURES

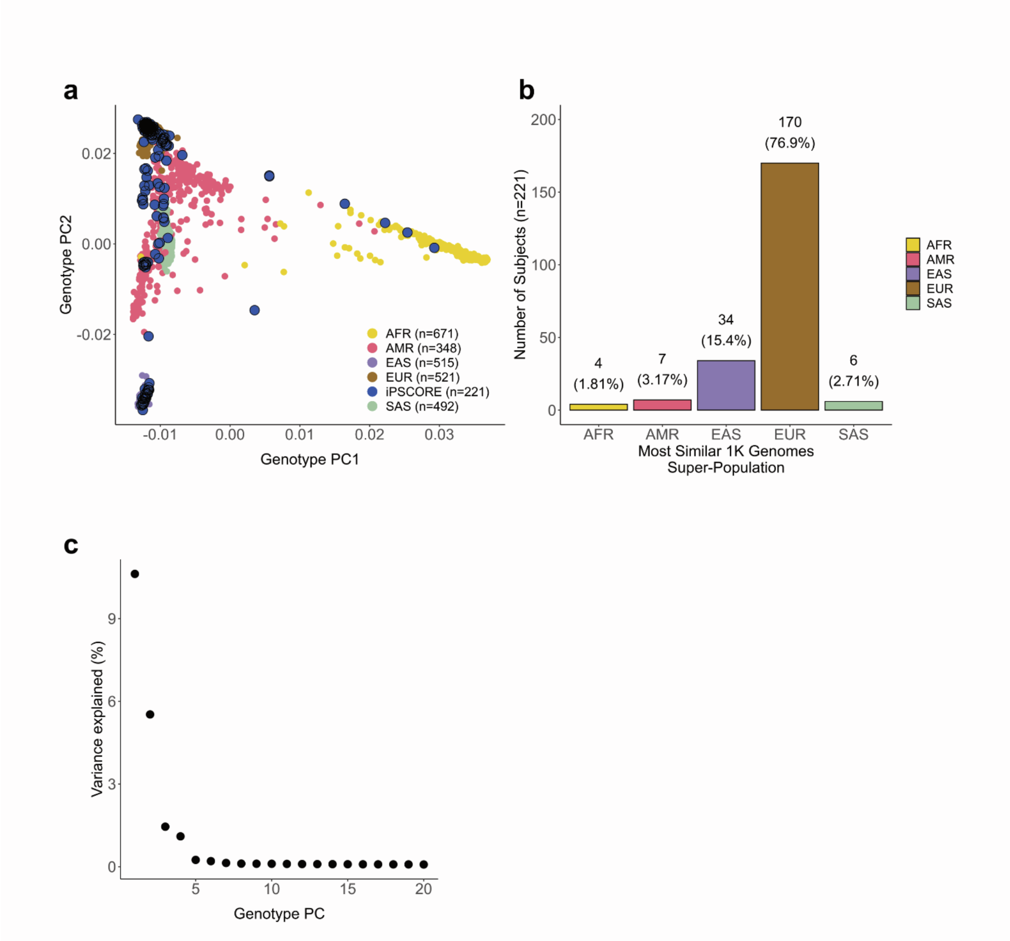

Figure S1. Genotype Principal Component Analysis (PCA) of iPSCORE Individuals, related to Figure 1.

a) Scatter plot showing the distribution of the 221 iPSCORE subjects (blue) included in this study, across the five super-populations defined in the 1000 Genomes. The x-axis represents genotype principal component 1, and the y-axis represents genotype principal component 2.

b) Distribution of the 221 iPSCORE subjects based on their closest matching super population as previously described[1].

c) Elbow plot showing the genotypic variance explained (y-axis) by each genotype principal component (x-axis). We found that genotype principal components 1-5 were sufficient to capture most of the genotypic variance explained by global ancestry and hence, will be used as covariates for QTL analyses.

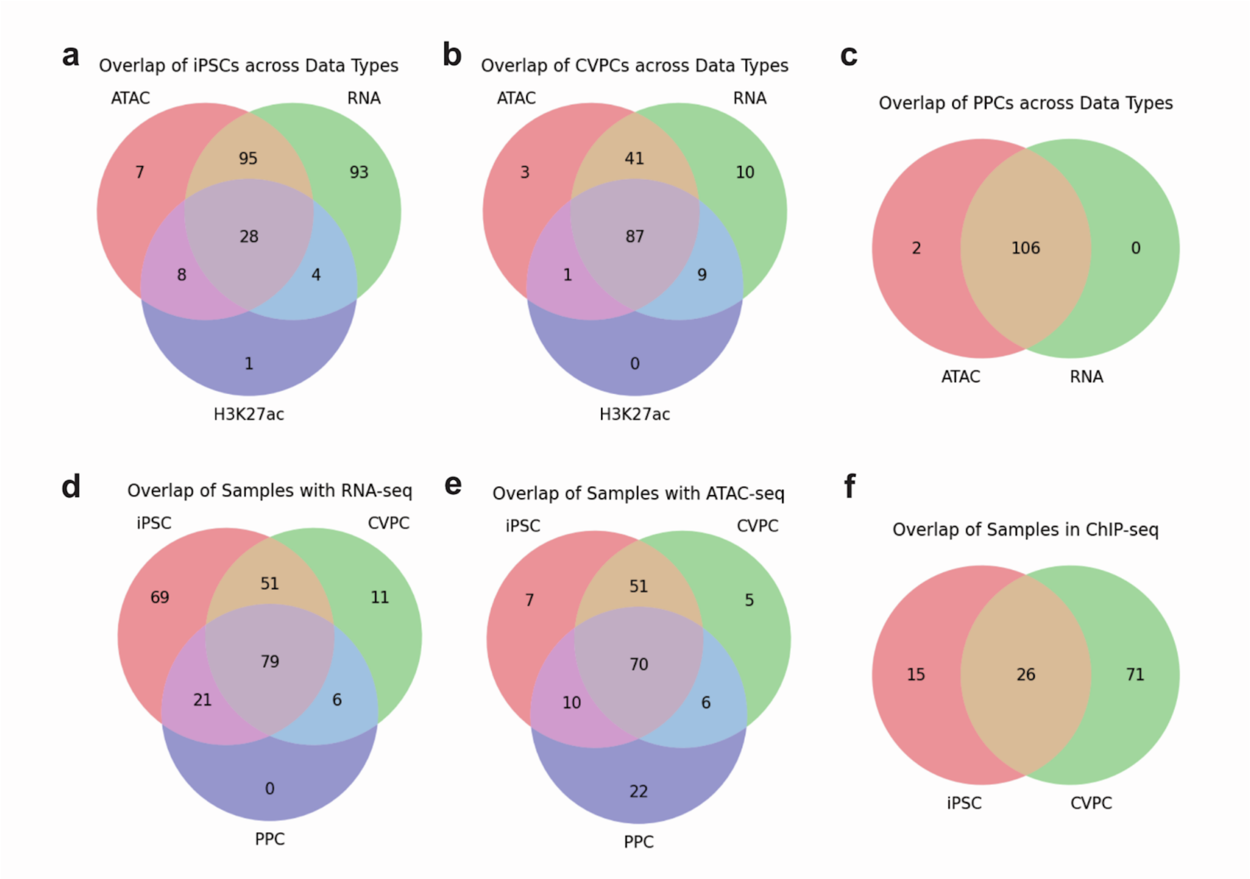

Figure S2. Overlap of Samples Across Molecular Data Types, related to Figure 1.

a) Venn diagram showing the overlap of iPSC lines ("iPSC_Line_ID" in Tables S2) across all data types. We note that only one iPSC line was used for each subject.

b) Venn diagram showing the overlap of CVPC differentiations ("UDID" in Tables S2) across all data types.

c) Venn diagram showing the overlap of PPC differentiations ("UDID" in Tables S2) across the data types.

d) Venn diagram showing the overlap of RNA-seq samples across the three tissue types based on iPSC line. For example, 79 iPSC lines from 79 subjects have RNA-seq for iPSC, CVPC, and PPC; 11 have RNA-seq for CVPC only; and 6 have RNA-seq for CVPC and PPC only.

e) Venn diagram showing the overlap of ATAC-seq samples across the three tissue types based on iPSC line. For example, 70 iPSC lines from 70 subjects have ATAC-seq for iPSC, CVPC, and PPC; 5 have ATAC-seq for CVPC only; and 6 have ATAC-seq for CVPC and PPC only.

f) Venn diagram showing the overlap of H3K27ac ChIP-seq samples between iPSC and CVPC. For example, 26 iPSC lines from 26 subjects have ChIP-seq for both iPSC and CVPC, and 71 have ChIP-seq for CVPC only.

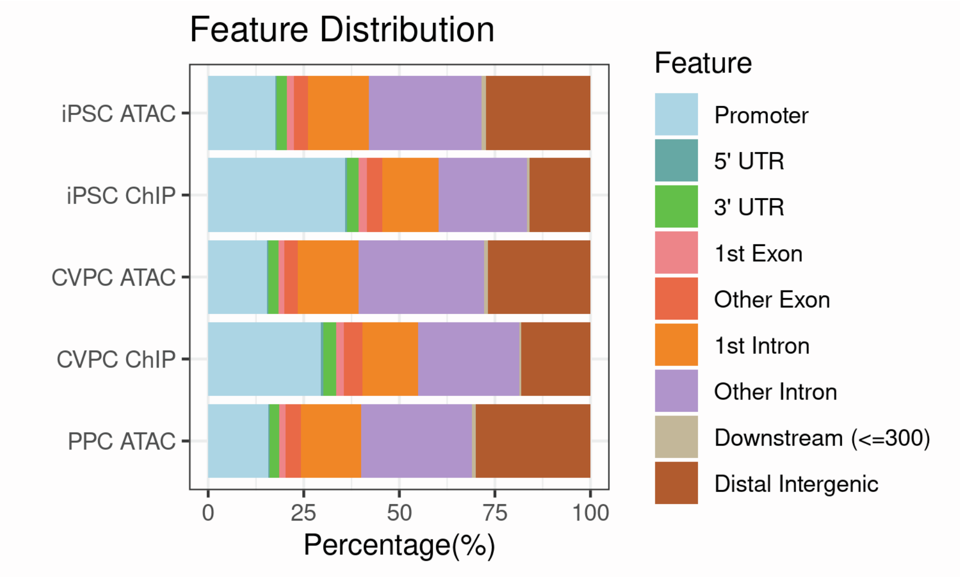

Figure S3. ATAC-seq and H3K27ac ChIP-seq Peak Annotations, related to Figure 2.

Bar charts showing the proportion of ATAC-seq and H3K27ac ChIP-seq peaks located in different functional regions. The x-axis is the percentage of peaks, the y-axis corresponds to the peak dataset (i.e. "Tissue" and "Data Type"), and the colors correspond to the genomic annotations assigned by ChIPseeker[2] using the UCSC hg38 gene coordinates.

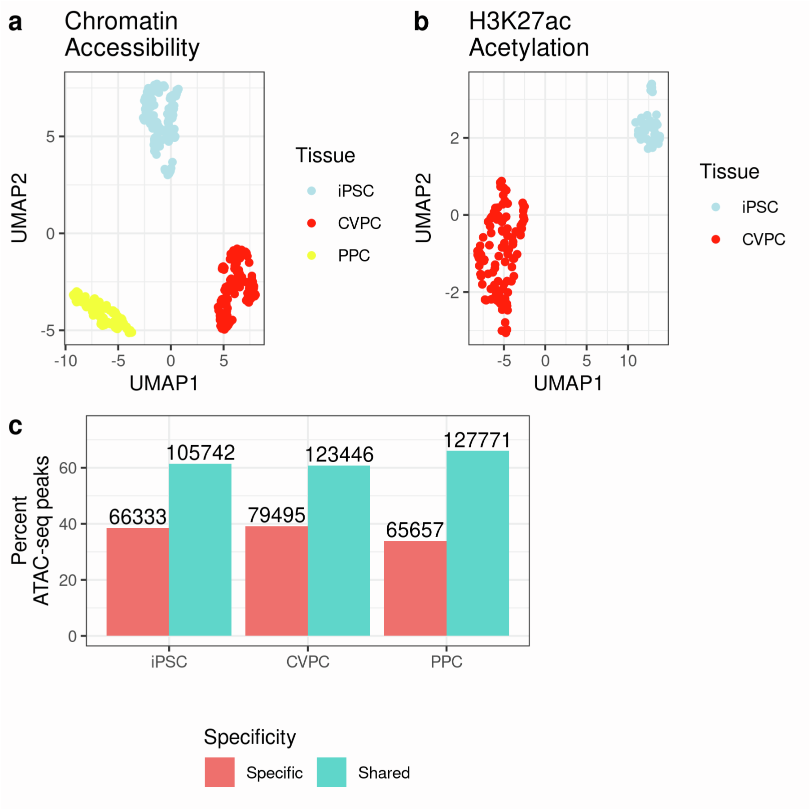

Figure S4. Characterization of iPSCORE Epigenomic Datasets, related to Figure 2.

a-b) UMAP of chromatin accessibility of 391 ATAC-seq **(a)** and histone acetylation of 144 H3K27ac ChIP-seq **(b)** samples from the iPSCORE Collection. Consensus peaks were curated for each dataset independently, and then merged to compare chromatin accessibility and histone acetylation across samples (See Methods). A UMAP analysis was performed using the top 2,000 most variable peaks. The samples cluster by tissue, indicating that the EDev-like iPSCs, CVPCs and PPCs each have distinct regulatory landscapes. Each point represents an ATAC-seq or ChIP-seq sample colored by their corresponding tissue.

c) Bar plot showing the percent of ATAC-seq peaks that are tissue-specific or shared. We intersected the three independent consensus ATAC-seq peak sets to annotate peaks that were specific to one tissue or shared between at least two tissues. Across all three EDev-like tissues, approximately one-third of ATAC-seq peaks were tissue-specific (range = 34-39.2%) and approximately two-thirds were shared (range = 60.8-66%). The bar colors correspond to tissue-specific (red) and shared (turquoise) peaks. The labels at the top of each bar correspond to the number of ATAC-seq peaks. The x-axis contains the tissue labels, and the y-axis is the percent of ATAC-seq peaks.

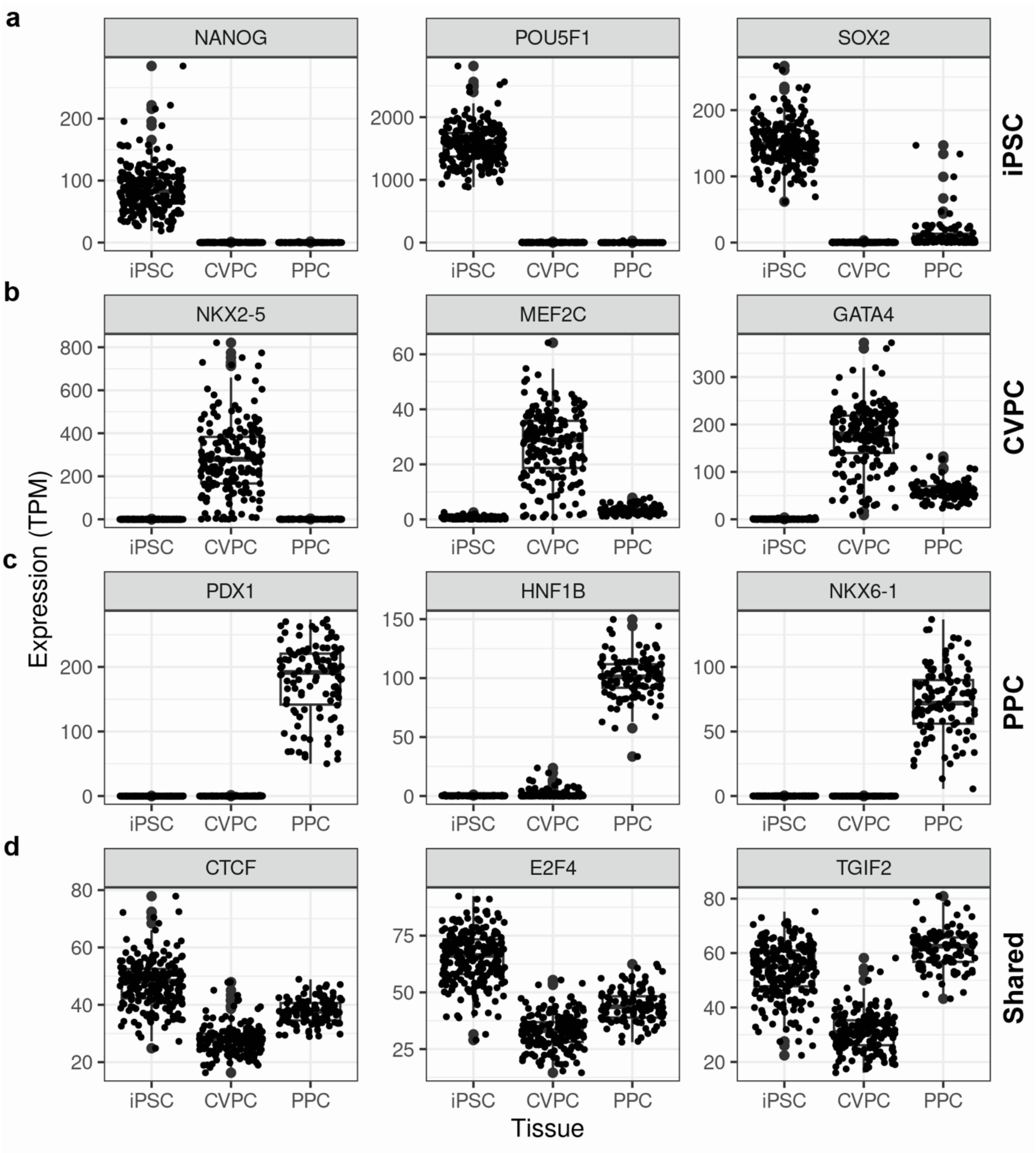

Figure S5. Expression of Tissue-specific TF Markers, related to Figure 2.

a-d) Boxplots showing the expression of tissue-specific iPSC **(a)**, CVPC **(b)**, PPC **(c),** and shared (**d**) TF markers. Each point represents an iPSCORE RNA-seq sample from the three tissues (x-axis) and the expression of the corresponding TFs (y-axis). The tissue-specific TF markers exhibit differential expression relative to the other tissues.

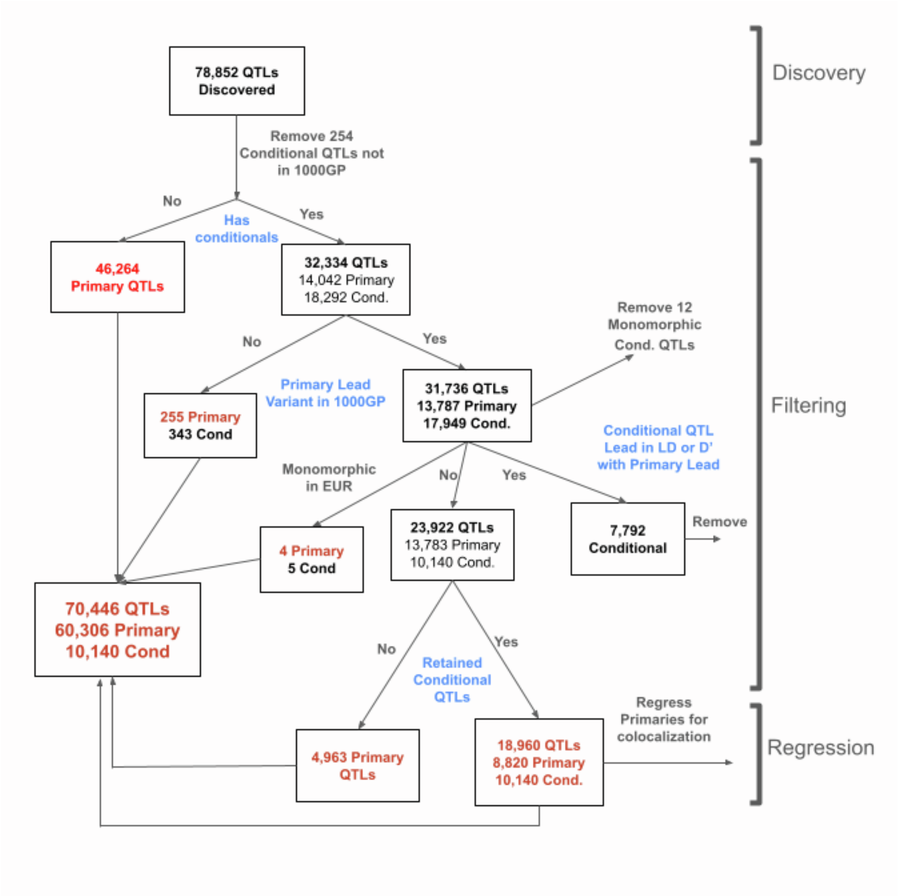

Figure S6. Overview of QTL Pipeline, related to Figure 2.

Multiple independent QTLs often affect the same element (i.e. gene or peak), however, primary QTL analyses only identify the strongest signal for each element, thus missing weaker independent signals[3]. In our study, we initially identified up to three conditional QTLs for each element by including the genotype of the top variant from the leading QTL as a covariate. This approach is commonly used to identify weaker independent QTLs by correcting for variants that are in linkage disequilibrium (LD) with the top variant(s) from the primary QTL(s). However, we observed that, while the lead variants from the conditional QTLs tended to not be in r^2 with the primary QTL lead variant, a large fraction were in high D', indicating that they are not independent genetic signals. Therefore, we added a filtering step to the QTL pipeline to remove non-independent conditional signals. The QTL pipeline consists of two steps: 1) QTL discovery, and 2) QTL Filtering. Step 1, QTL discovery, is standard practice and calculates up to four loci (primary and three conditional) for a given qElement and reports the lead variant for each locus. Step 2, QTL Filtering, identifies and removes conditional QTLs with lead variants in high D 'with the primary QTL lead variant (Table S4).

Step 1: QTL Discovery

(1) **Primary QTL**: gene expression ~ SNP gt + covariates + kinship -> Lead Variant = SNP_A
(2) **Conditional1 QTL**: gene expression ~ SNP gt + covariates + gt_{SNP_A} + kinship -> Lead Variant = SNP_B
(3) **Conditional2 QTL**: gene expression ~ SNP gt + covariates + gt_{SNP_A} + gt_{SNP_B} + kinship -> Lead Variant = SNP_C

In the figure, we depict the QTL pipeline: Step 1, QTL Discovery identified 78,852 QTLs for 60,306 unique qElements with a primary QTL across the eight datasets. Within the flow chart, QTLs in red were retained. In Step 2 QTL Filtering, to filter conditional QTLs with lead variants in LD (D' $\geq$ 0.8 and/or $r^2 \geq 0.8$) with their corresponding primary QTL lead variants, required that the conditional lead variants be present in the 1000 Genomes Project EUR population, hence 254 conditional QTLs were removed. After this initial filtering, 46,264 qElements only had a primary QTL signal, while 14,042 qElements had conditional signals. Of these 14,042 qElements, 255 had primary QTLs with lead variants that are not present in the 1000 Genomes EUR population. In these cases, we cannot determine the relationship between the primary lead variant and the conditional lead variant(s); therefore, we retained the 255 primary QTLs associated with these qElements and removed the 343 conditional QTLs. We retained the 255 primary QTLs even though their lead variants were not in the 1000 Genomes Project EUR population because downstream analyses, such as GWAS colocalization, do not require the QTL lead variant to be present to calculate the posterior probabilities for the remaining SNPs in the loci.

After the above filtering, we retained 13,787 qElements with both primary and conditional QTL lead variants in the 1000 Genomes Project EUR population. We next calculated the LD and D' between the 17,949 conditional QTL lead variants and their corresponding 13,787 primary QTL lead variant in the 1000 Genomes EUR population, using *plink*[4]. We identified 7,792 non-independent conditional QTLs ($r^2 \geq 0.8$ and/or D' $\geq$ 0.8), 4 primary QTLs with monomorphic lead variants (in the 1000 Genomes Project EUR population) with 5 associated conditional QTLs, and 12 conditional QTLs with monomorphic lead variants. We removed the 7,792 non-independent conditional QTLs, 12 monomorphic conditional QTLs, and the 5 conditional QTLs associated with monomorphic primary QTLs. This resulted in 4,963 qElements that lost all associated conditional QTLs and 8,820 qElements with 10,140 conditional QTLs. The 8,820 primary QTLs with conditional QTLs were regressed prior to GWAS colocalization.

QTL Filtering (Step 2), resulted in 70,446 QTLs including 60,306 primary (51,486 non-regressed and 8,820 regressed) and 10,140 conditional. We characterize the 60,306 primary and 10,140 conditional QTLs after the filtering step in Figure 2b-d; use the 60,306 primary lead variants in characterization analyses (Figure 2e; Figure 3; Figure 4); and use the 60,306 primary QTLs for GWAS colocalization (Figure 5; Figure 6). See Figure S10 for the QTL regression Step.

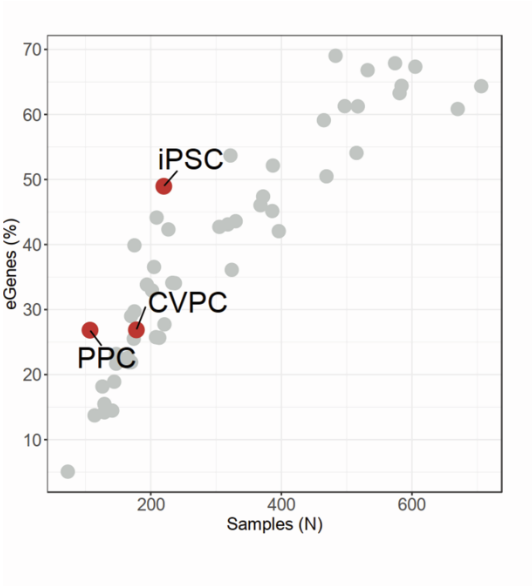

Figure S7. eGene Discovery Rate, related to Figure 2.

Scatter plot showing the percentage of expressed genes that are eGenes in each tissue relative to the 49 GTEx tissues (including 47 adult and 2 cell culture tissues; version 8), as a function of sample size. These findings support that the eGene discovery rate in the three iPSCORE EDev-like tissues is similar to the 49 tissues in the GTEx Consortium[5]. Gray points represent GTEx adult tissues while red points represent the three iPSCORE EDev-like tissues in this study.

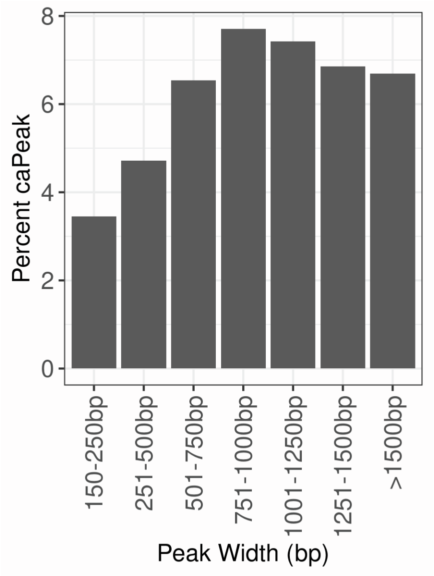

Figure S8. caQTL Discovery Rate by Peak Width, related to Figure 2.

Bar plots showing the caQTL discovery rate by ATAC-seq peak width. caQTL discovery rates were associated with the ATAC-seq peak width. 7.9% peaks with widths between 751-1000 bp had caQTLs, while only 3.7% of shorter peaks (151-300 bp) had caQTLs. Shorter ATAC-seq peaks tend to have fewer mapped reads, therefore it is unclear whether this observation is driven by biological or technical factors, still it provides useful information for establishing caQTL pipeline.

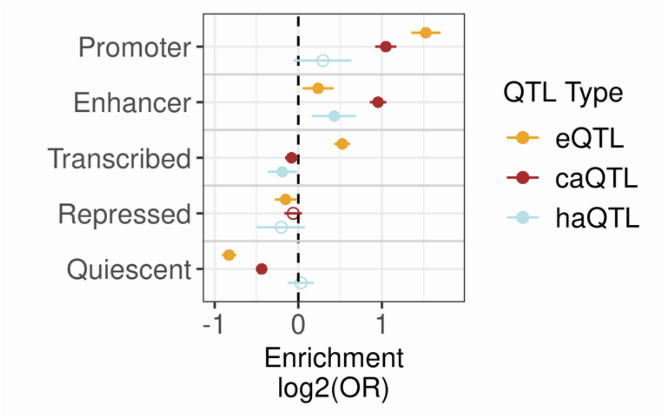

Figure S9. iPSC QTL Chromatin State Enrichment, related to Figure 2.

Plot showing the enrichment of iPSC primary eQTLs, caQTLs, and haQTLs in chromatin states. The x-axis is the enrichment $\log_2$(Odds Ratio) and the y-axis contains the five collapsed chromatin states. The points are colored by the QTL type (eQTL = "orange", caQTL = "brown", and haQTL = "light blue"). The whiskers represent the $\log_2$ upper and lower 95% confidence intervals. Significant enrichments are represented by filled circles and non-significant enrichments are represented by circles without a fill.

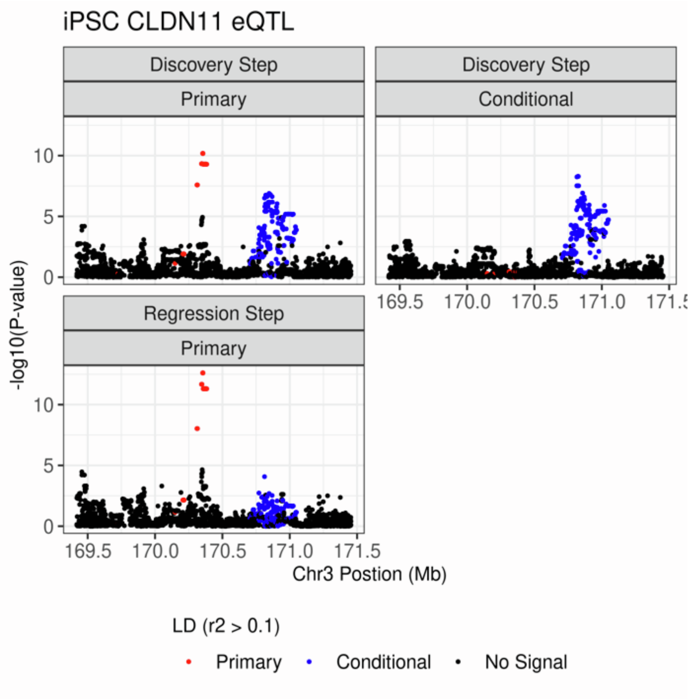

Figure S10. Primary QTL Regression, related to Figure 5.

In the QTL Discovery pipeline step 1, the primary QTLs were not conditioned, thus did not account for the presence of multiple independent loci (Figure S6). Therefore, prior to using Bayesian colocalization to identify the overlap of iPSCORE QTLs and loci from 15 GWAS studies, we regressed 8,820 primary QTL signals (see Figure S6) to remove the effects of their corresponding 10,140 conditional QTL signals.

Regressed Primary eQTL: gene expression ~ SNP gt + covariates + gt_{SNP_B} + gt_{SNP_C} + kinship

The inclusion of Primary QTL Regression resulted in cleaner primary QTL signals (Figure S10). Collectively, our two-step QTL pipeline and primary QTL regression improved the interpretation of GWAS colocalization by removing the effect of the conditional signals on the primary QTL and showed that a large fraction (43%) of conditional QTLs are not independent because they are in high LD and/or D' with the primary lead variant.

In Figure S10, we show an example to illustrate the affects of regression: Manhattan plots of the primary and conditional iPSC *CLDN11* eQTL signals before regression (top row) and the primary iPSC *CLDN11* eQTL signal after regression (bottom left). The genomic coordinates are on the x-axes, the -$\log_{10}$(P-values) for the associations between the genotype of the tested variants and *CLDN11* expression are plotted on the y-axes, each point represents a variant. Variants are colored

by signal, where the variants in the primary signal are red, variants in the conditional signal are blue, and variants not associated with a signal are black. Prior to regression, the conditional signal (in blue) is present between 170.5 and 171Mb in the primary QTL (top left), while after regression, the conditional signal (in blue) is corrected for and therefore not present (bottom left), leaving just the primary signal (in red).

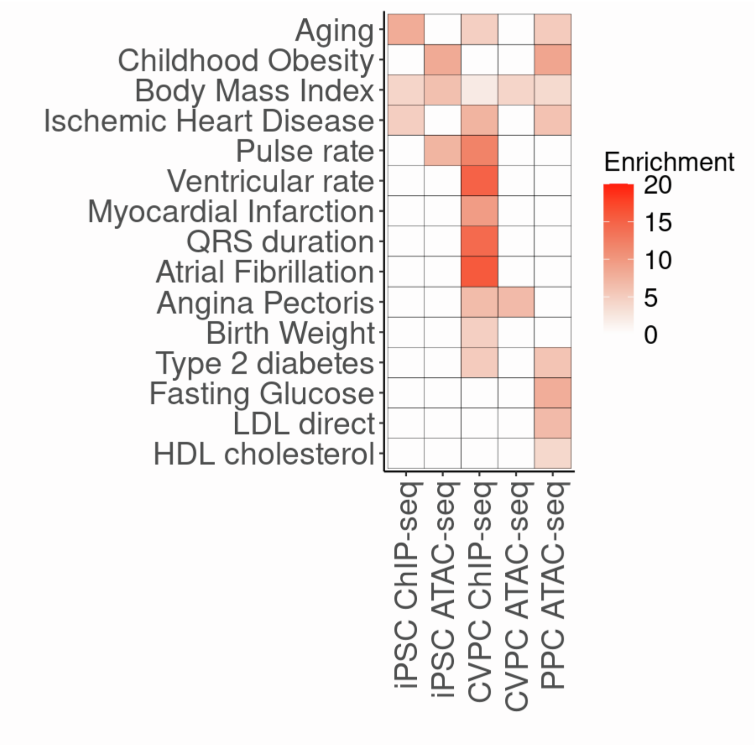

Figure S11. Enrichment of GWAS in ATAC-seq, related to Figure 5.

Heatmap showing the enrichment of GWAS variants in ATAC-seq and H3K27ac ChIP-seq peaks from all three tissues. Enrichment (the ratio of the proportion of heritability explained by the annotation and the proportion of SNPs in the annotation) of GWAS variants in the peaks was calculated using LD Score regression. The y-axis corresponds to the 15 summary statistics and the x-axis corresponds to the five tested peak sets. Each cell is filled with the LD Score regression enrichment. For plot legibility, the maximum enrichment was set to 20 and non-significant (P-value > 0.01) tests were filled white.

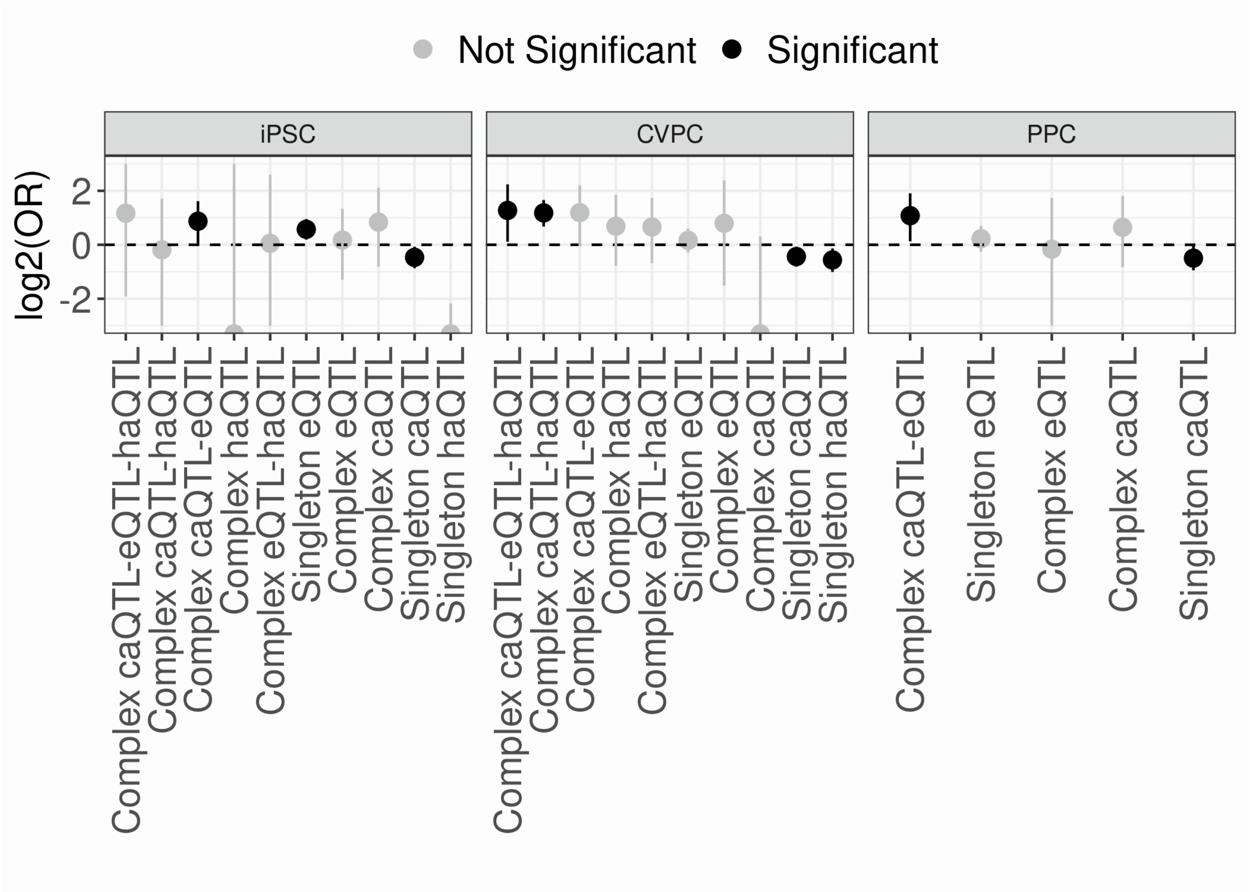

Figure S12: Enrichment of GWAS Colocalization by QTL type and Tissue, related to Figure 5. Plots showing the relative enrichment of GWAS colocalization by complex QTL and singleton composition for each the three EDev-like tissues. For each tissue, we performed two-sided Fisher's Exact Tests to test the relative enrichment (odds ratio) of each QTL category for GWAS colocalization compared to all other categories. From left to right, the panels correspond to the iPSC, CVPC, and PPC enrichments. The points represent the $\log_2$(Odds Ratio) for each enrichment and the whiskers represent the $\log_2$ of the 95% confidence intervals. Tests that had P-value < 0.05 were considered significant (colored in black).

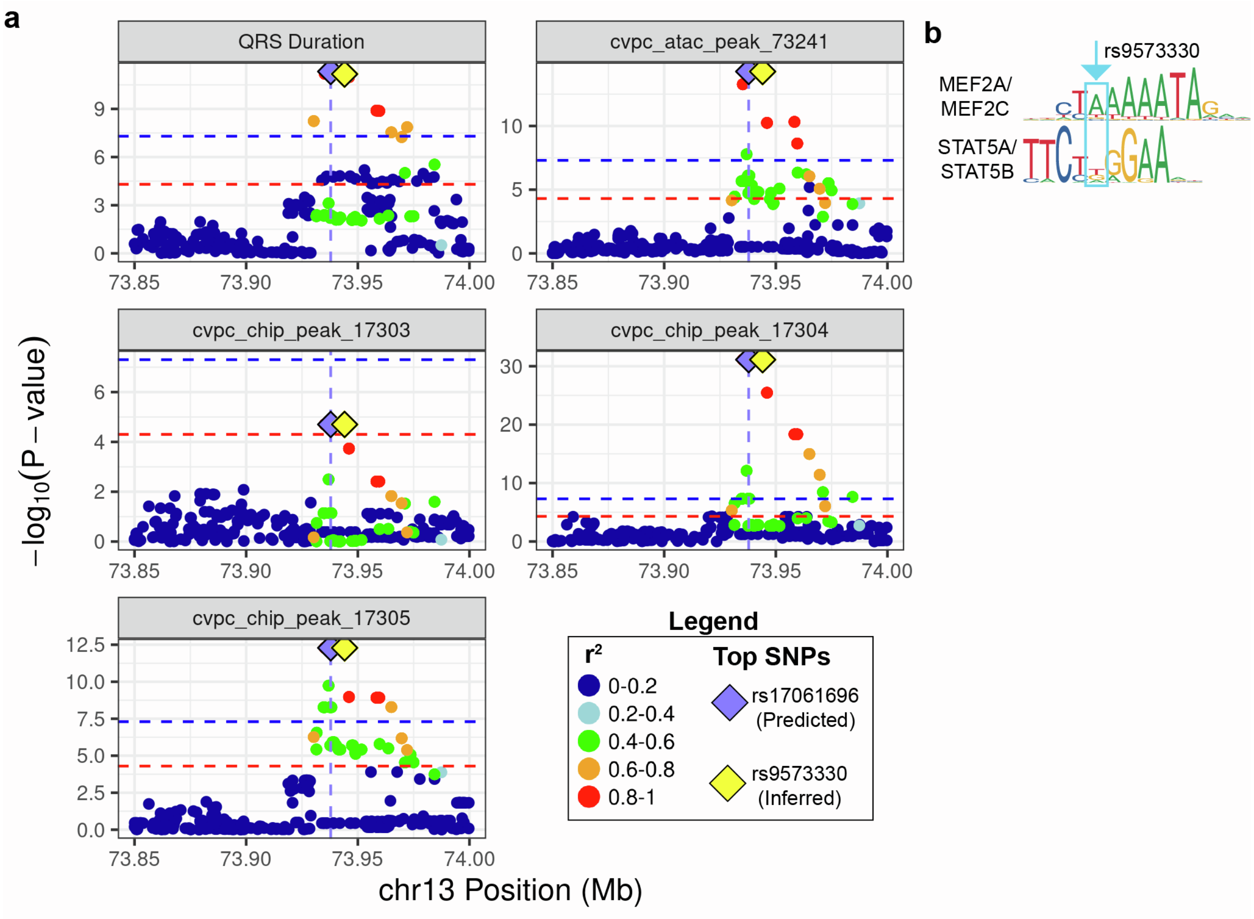

Figure S13: Identification of QRS Duration KLF12 Locus with Multiomic QTLs, related to Figure 6.

a) A QRS Duration signal colocalized with CVPC complex QTL 274 containing one caPeak and three haPeaks. The genomic coordinates are on the x-axes, and the -$\log_{10}$(P-values) for the associations between the genotype of the tested variants and chromatin accessibility, H3K27 acetylation or QRS duration are plotted on the y-axes. Horizontal lines indicate genome-wide significance thresholds for QTL (P-value = $5x10^{-5}$; red) and GWAS (P-value = $5x10^{-8}$; blue) for plotting purposes. Each variant was colored according to their LD with the lead fine-mapped variant (purple diamond; rs17061696, chr13:73937854:G>C, causal PP = 31.8%) using the 1000 Genomes Phase 3 Panel (Europeans only) as reference. rs9573330 (chr13:73944073:G>A, yellow diamond) is a MOPCV and the likely causal variant.

b) Binding site motifs for MEF2A/MEF2C and STAT5A/STAT5B overlap the validated MOPCV (chr13:73944073:G>A; rs9573330) for QRS duration. The light blue box and arrow indicates

which position in the motifs is overlapped by rs9573330. MEF2A and MEF2C have a high degree of sequence similarity, therefore only MEF2A is shown for plot legibility.

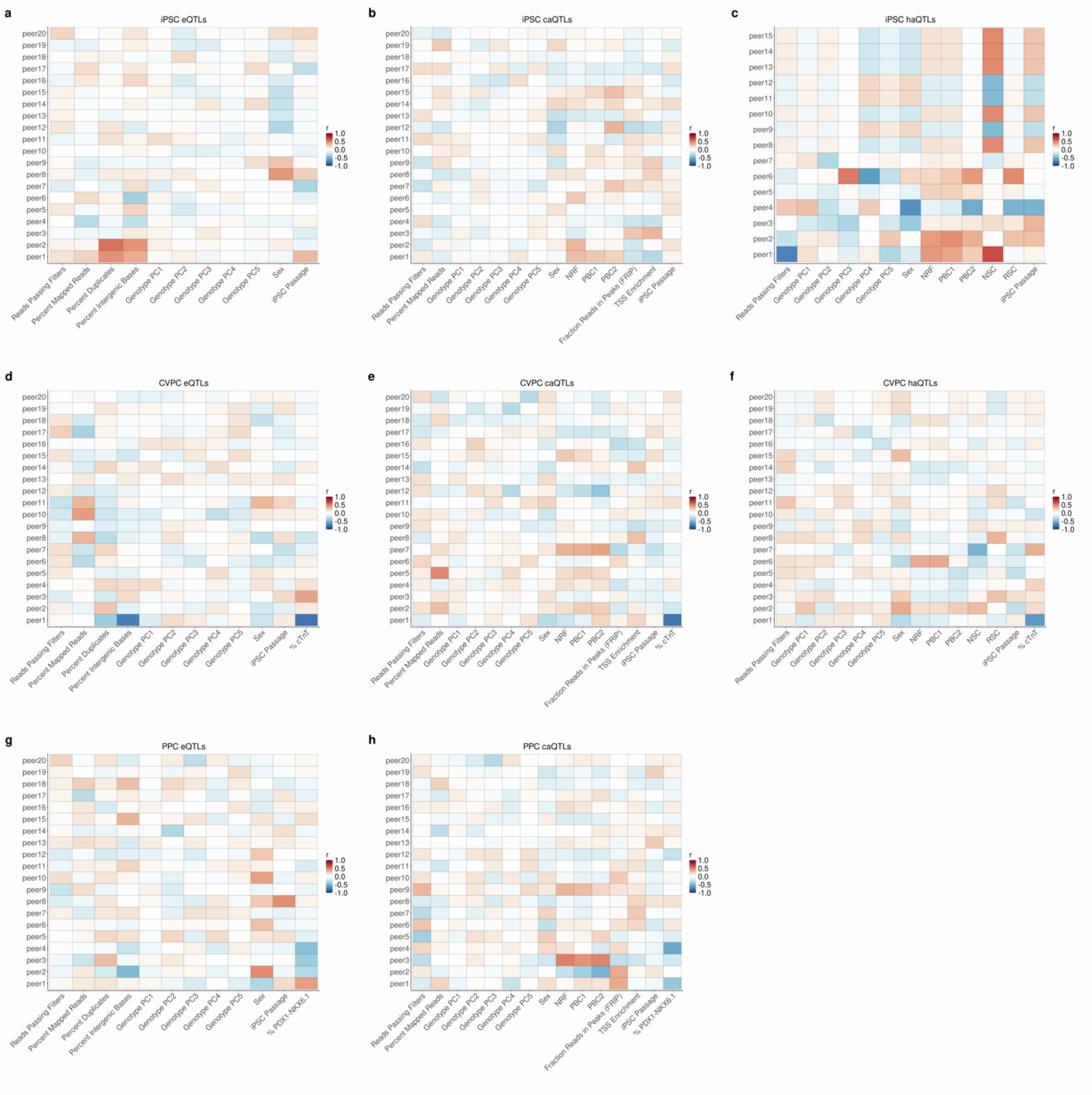

Figure S14. PEER Factor Correlation with Known Covariates, related to the STAR Methods. Heatmaps showing the Pearson correlation (r) between PEER factors and the known covariates for each tissue type. First row shows the correlation with PEER factors calculated for eQTLs. Second row shows the correlation with PEER factors calculated for caQTLs. Third row shows the correlation with PEER factors calculated for haQTLs. “Reads passing filters” is the number of reads passing filters. “Genotype PC1-5” are the genotype principal components capturing global ancestry. “Percent Duplicates” is the percentage of duplicate reads. “Percent Intergenic Bases” is the percentage of bases that mapped to intergenic regions. “iPSC Passage" is the passage of the

iPSCs before CVPC or PPC differentiation. For iPSCs, this indicates the passage of iPSCs upon cell harvest. For CVPCs, "% cTnT" is the percentage of cells positive for cardiac troponin detected by flow cytometry. For PPCs, "% PDX1-NKX6.1" is the percentage of double-positive $PDX1^{+}NKX6\text{-}1^{+}$ cells detected by flow cytometry. These findings show that the inclusion of PEER factors in the QTL mapping linear model accounts for cell type heterogeneity in CVPCs and PPCs. "NRF" is the non-redundant fraction of reads (i.e., fraction of distinct uniquely mapping reads). "PBC1" is the PCR bottleneck coefficient 1. "PBC2" is the PCR bottleneck coefficient 2. "NSC" is the normalized strand cross-correlation coefficient. "RSC" is the relative strand cross-correlation coefficient. These results show that the top 1-3 PEER factors were correlated with sequencing quality, differentiation efficiency and sex.

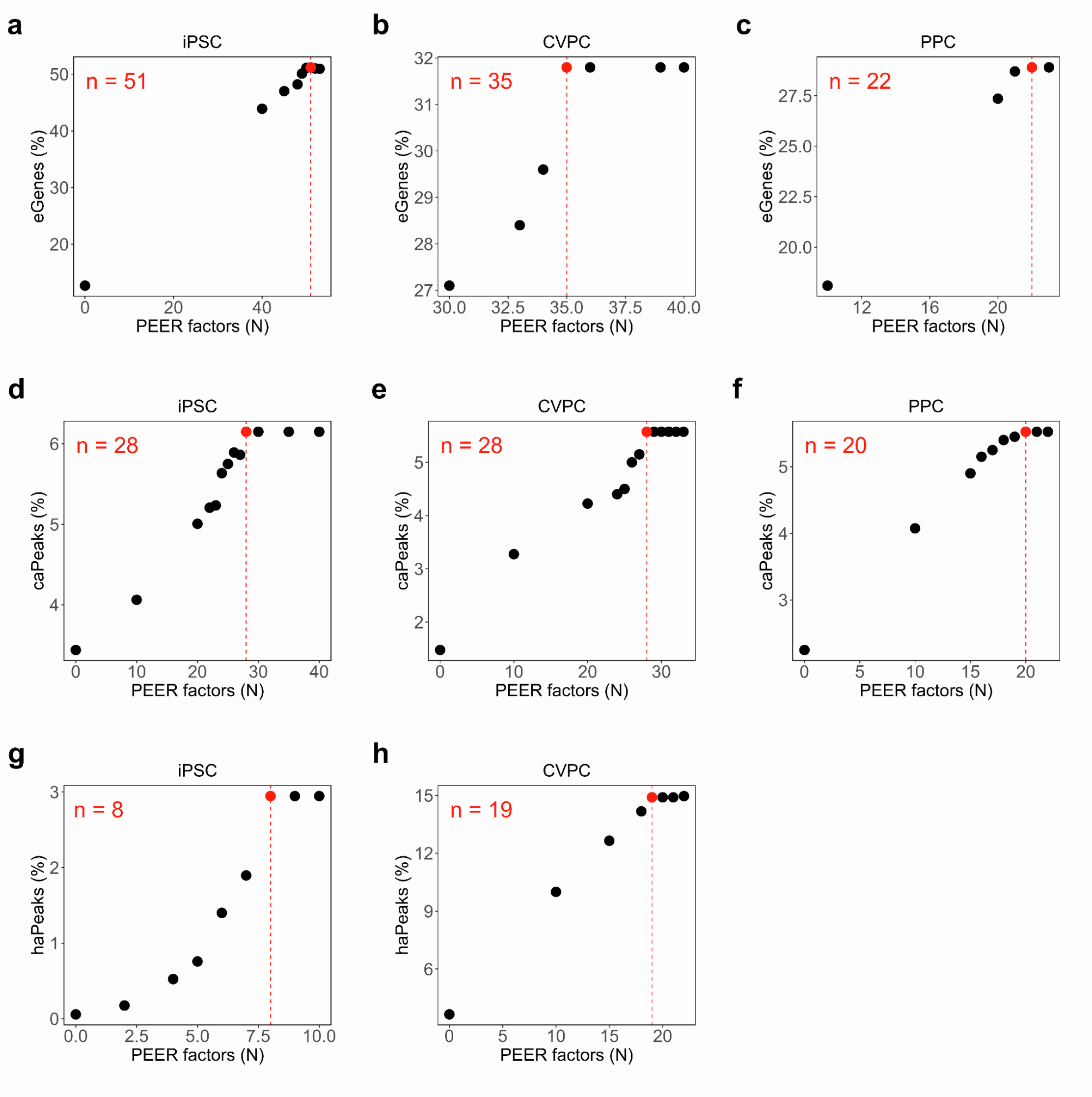

Figure S15. PEER Factor Optimization, related to STAR Methods.

Point plots showing the percentage of qElements (eGenes, caPeaks, haPeaks) that were discovered with varying numbers of PEER factors as covariates. The top row shows PEER optimization results for eQTLs, the middle row shows results for caQTLs, and the bottom row shows results for haQTLs. Red indicates the least number of PEER factors that resulted in maximum eGene/caPeak/haPeak discovery.

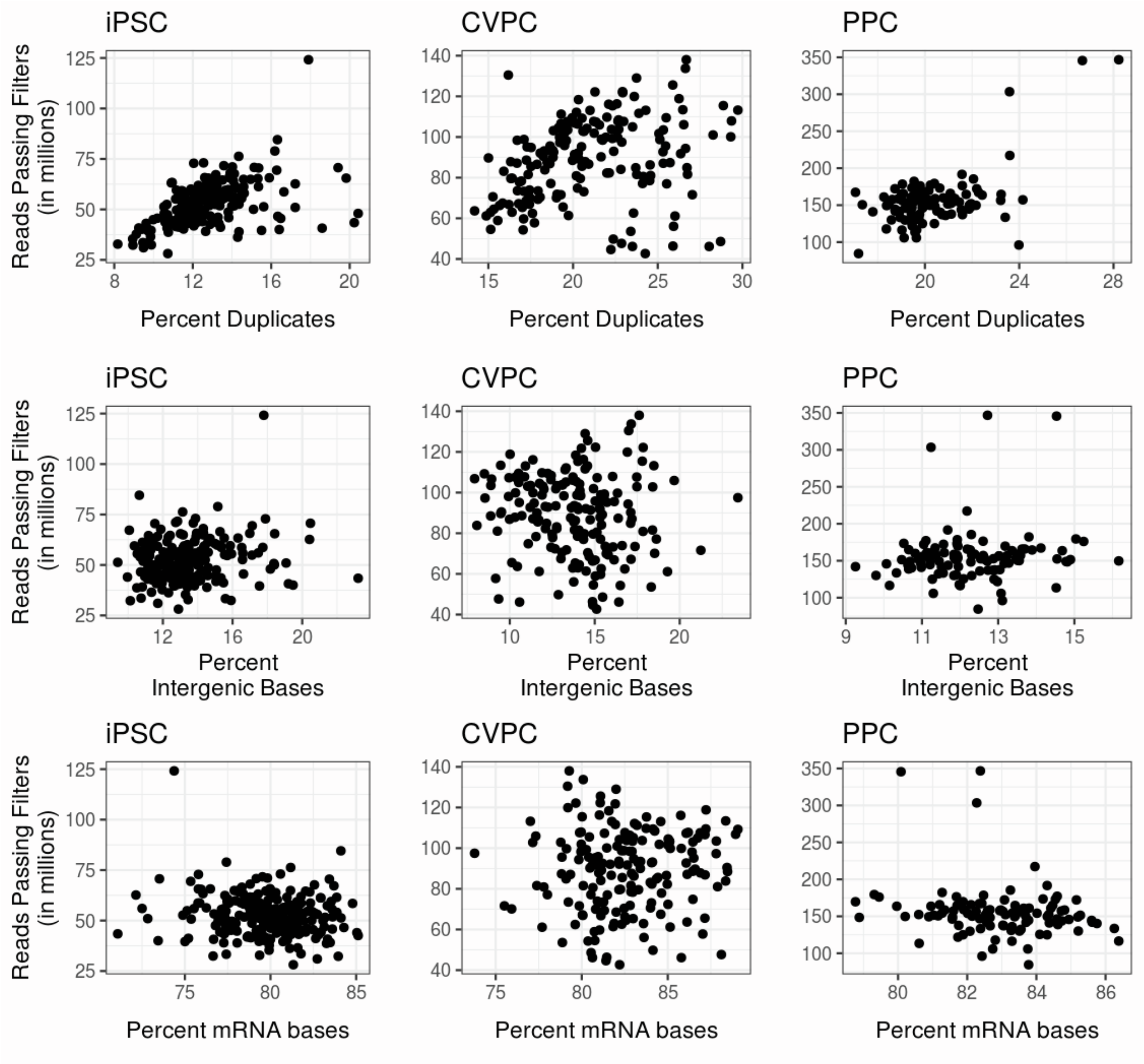

Figure S16. RNA-seq Quality Control, related to STAR Methods.

Scatter plots displaying the quality of the RNA-seq samples from iPSCs (n = 220), CVPCs (n = 178), and PPCs (n = 107). The plots show the number of reads passing filters (y-axis) against the percent of duplicate reads (row 1), the percent of intergenic bases (row 2), and the percent mRNA bases (row 3) for all 505 RNA-seq samples. All samples have been published in previous iPSCORE eQTL publications[1,6,7], with the exception of 7 iPSC samples that were newly incorporated into this study.

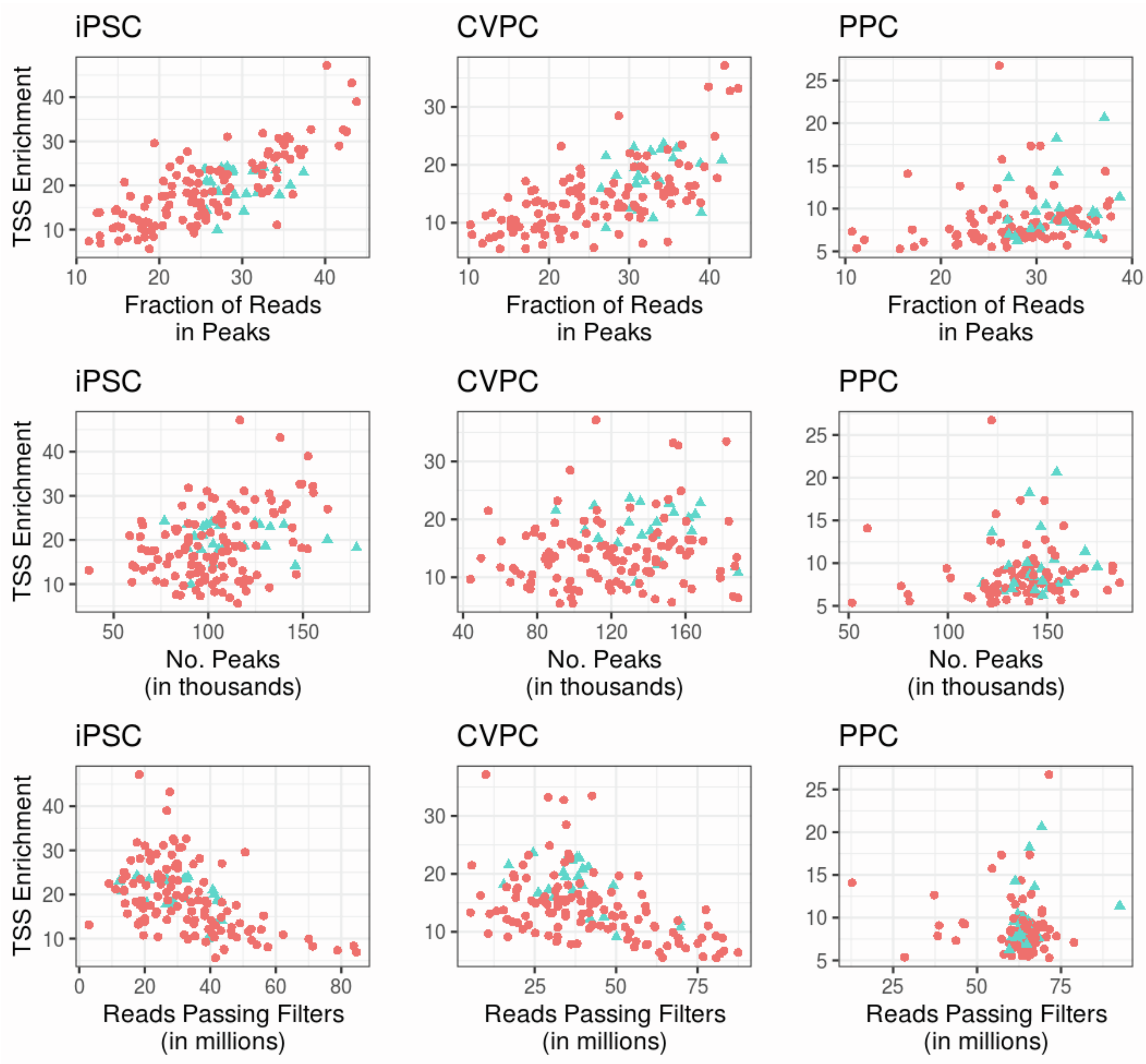

Figure S17. ATAC-seq Quality Control and Reference Sample Selection, related to STAR Methods.

Scatter plots displaying the quality of the ATAC-seq samples from all three tissues. The top row shows the transcription start site enrichment (TSSE; calculated by the ATACseqQC R package[8]) of the samples plotted against the fraction of reads in peaks (FRiP), the middle row shows the TSSE and the number of ATAC-seq peaks per sample, and the bottom row shows the TSSE and the number of reads passing filters. We selected reference samples (blue triangles) based on QC metrics (See Methods) from unrelated individuals for each tissue (iPSC n=24; PPC n=24; and

CVPC n=23) to establish a set of consensus peaks for the quantification of chromatin accessibility across the respective tissues.

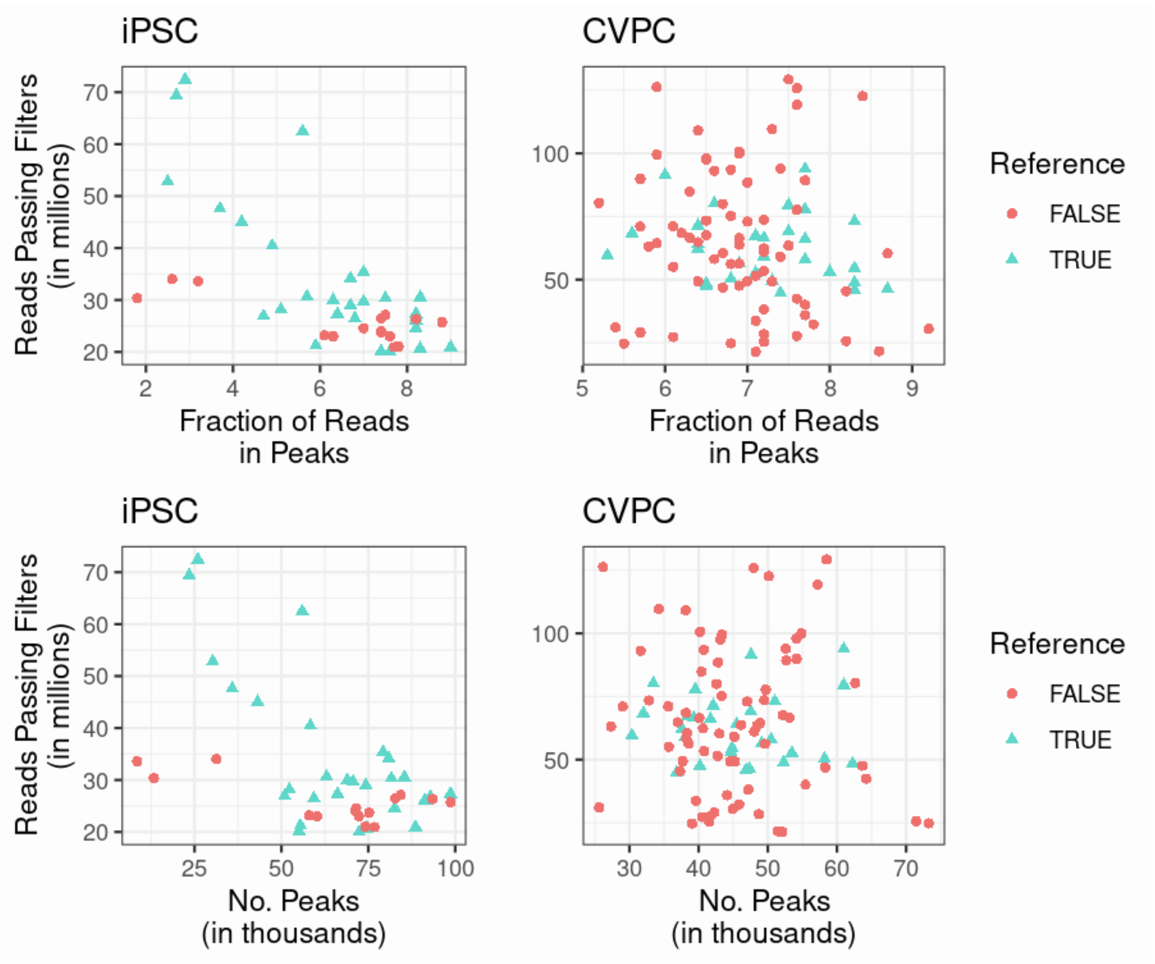

Figure S18. H3K27ac ChIP-seq Quality Control and Reference Sample Selection, related to STAR Methods.

Scatter plots displaying the quality of the H3K27ac ChIP-seq samples from iPSCs (n = 43) and CVPCs (n =101). The top row shows the number of reads passing filters of the samples plotted against the fraction of reads in narrow peaks (FRiP), and the bottom row shows the number of reads passing filters against the number of peaks. We selected reference samples (blue triangles) based on QC metrics (see Methods) from unrelated individuals for each tissue (iPSC n = 28; and CVPC n = 29) to establish a set of consensus peaks for the quantification of chromatin accessibility across the respective tissues.

SUPPLEMENTAL REFERENCES

1. Panopoulos, A.D., D'Antonio, M., Benaglio, P., Williams, R., Hashem, S.I., Schuldt, B.M., DeBoever, C., Arias, A.D., Garcia, M., Nelson, B.C., et al. (2017). iPSCORE: A Resource of 222 iPSC Lines Enabling Functional Characterization of Genetic Variation across a Variety of Cell Types. Stem Cell Reports *8*, 1086–1100. https://doi.org/10.1016/j.stemcr.2017.03.012.

2. Yu, G., Wang, L.-G., and He, Q.-Y. (2015). ChIPseeker: an R/Bioconductor package for ChIP peak annotation, comparison and visualization. Bioinformatics *31*, 2382–2383. https://doi.org/10.1093/bioinformatics/btv145.

3. Jansen, R., Hottenga, J.-J., Nivard, M.G., Abdellaoui, A., Laport, B., de Geus, E.J., Wright, F.A., Penninx, B.W.J.H., and Boomsma, D.I. (2017). Conditional eQTL analysis reveals allelic heterogeneity of gene expression. Hum Mol Genet *26*, 1444–1451. https://doi.org/10.1093/hmg/ddx043.

4. Purcell, S., Neale, B., Todd-Brown, K., Thomas, L., Ferreira, M.A.R., Bender, D., Maller, J., Sklar, P., de Bakker, P.I.W., Daly, M.J., et al. (2007). PLINK: a tool set for whole-genome association and population-based linkage analyses. Am J Hum Genet *81*, 559–575. https://doi.org/10.1086/519795.

5. GTEx Consortium (2020). The GTEx Consortium atlas of genetic regulatory effects across human tissues. Science *369*, 1318–1330. https://doi.org/10.1126/science.aaz1776.

6. D'Antonio, M., Nguyen, J.P., Arthur, T.D., iPSCORE Consortium, Matsui, H., D'Antonio-Chronowska, A., and Frazer, K.A. (2023). Fine mapping spatiotemporal mechanisms of genetic variants underlying cardiac traits and disease. Nat Commun *14*, 1132. https://doi.org/10.1038/s41467-023-36638-2.

7. Nguyen, J.P., Arthur, T.D., Fujita, K., Salgado, B.M., Donovan, M.K.R., iPSCORE Consortium, Matsui, H., Kim, J.H., D'Antonio-Chronowska, A., D'Antonio, M., et al. (2023). eQTL mapping in fetal-like pancreatic progenitor cells reveals early developmental insights into diabetes risk. Nat Commun *14*, 6928. https://doi.org/10.1038/s41467-023-42560-4.

8. Ou, J., Liu, H., Yu, J., Kelliher, M.A., Castilla, L.H., Lawson, N.D., and Zhu, L.J. (2018). ATACseqQC: a Bioconductor package for post-alignment quality assessment of ATAC-seq data. BMC Genomics *19*, 169. https://doi.org/10.1186/s12864-018-4559-3.
